# Bioelectric Responses of Conjunctival Goblet Cells to Dry Eye: Impact of Ion Channels on Exocytotic Function and Viability

**DOI:** 10.3390/ijms21249415

**Published:** 2020-12-10

**Authors:** Donald G. Puro

**Affiliations:** 1 Department of Ophthalmology & Visual Sciences, University of Michigan, 1000 Wall Street, Ann Arbor, MI 48105, USA; dgpuro@umich.edu; 2 Department of Molecular & Integrative Physiology, University of Michigan, 1000 Wall Street, Ann Arbor, MI 48105, USA

**Keywords:** hyperosmolarity, K_ATP_ channels, nonspecific cation channels, voltage-gated calcium channels, P2X_7_ receptor/channels, exocytosis, membrane capacitance, death

## Abstract

How ion channels impact the response of the ocular surface to dry eye is only beginning to be explored. Here, we review recent progress and provide new experimental data clarifying the exocytosis-altering actions of ion channels in conjunctival goblet cells whose release of tear-stabilizing mucin is a key adaptive response to the pre-ocular hyperosmolarity that characterizes dry eye. Patch-clamp recordings of goblet cells located in freshly excised rat conjunctiva reveal that these mucin-releasing cells respond to sustained hyperosmolarity by sequentially activating their ATP-sensitive potassium (K_ATP_), nonspecific cation (NSC), voltage-gated calcium (VGCC), and P2X_7_ channels; each of which modulates exocytosis. Based on these and other new findings, we now identify four stages in the bioelectric response of conjunctival goblet cells to extracellular hyperosmolarity. To better characterize these stages, we report that high-resolution membrane capacitance (Cm) measurements of the exocytotic activity of single goblet cells demonstrate that the replenishment of mucin-filled granules after neural-evoked exocytosis is a multi-hour process, which VGCCs markedly accelerate. Yet, we also discovered that VGCC activation is high-risk since hyperosmotic-induced goblet cell death is boosted. With dry eye treatments being far from optimal, elucidating the physiologic and pathobiologic impact of the K_ATP_/NSC/VGCC/P2X_7_ pathway provides a new opportunity to identify novel therapeutic strategies.

## 1. Introduction

Dry eye is one of the most common disorders affecting the visual system. The hallmark of this uncomfortable sight-threatening condition is the accelerated evaporation of the pre-ocular tear film [1] whose integrity is essential for optimal vision. Despite the multitude of etiologies for clinical dry eye, a universal feature is the evaporation-induced increase in tear osmolarity to levels above the normosmotic ~300 mosM [1]. Although correlation between the magnitude of tear hyperosmolarity and the severity of reported symptoms can vary, discomfort and blurred vision are typically greater at higher osmolarities, which may reach 360 mosM or more in subjects with dry eye [1,2]. While it is well-established that pre-ocular dryness/hyperosmolarity triggers many complex biologic responses, including inflammatory processes that contribute to the pathogenesis of chronic dry eye [3,4,5], much remains to be learned about the mechanisms by which the ocular surface system responds to the onset of dry eye-induced hyperosmolarity.

With the tear film providing ~70% of the refractive power needed to achieve excellent vision [6], it is not surprising that the ocular surface is highly adapted to rapidly respond to disruption of this complex fluid layer. A key adaptive component of the ocular surface system is the conjunctival goblet cell whose exocytotic release of tear-stabilizing mucin is evoked by neural reflexes triggered by pre-ocular dryness/hyperosmolarity [2,7,8]. Recently, our patch-clamp studies of goblet cells located within freshly excised specimens of the rat conjunctiva revealed that sustained hyperosmolarity results in the sequential activation of ATP-sensitive K^+^ (K_ATP_) channels, nonspecific cation (NSC) channels, and voltage-gated calcium channels (VGCCs), as well as purinergic P2X_7_ receptor/channels [9,10]. Further, we discovered that this K_ATP_/NSC/VGCC/P2X_7_ pathway robustly modulates the neural-evoked exocytosis of mucin-filled granules [9,10].

**Figure 1:**
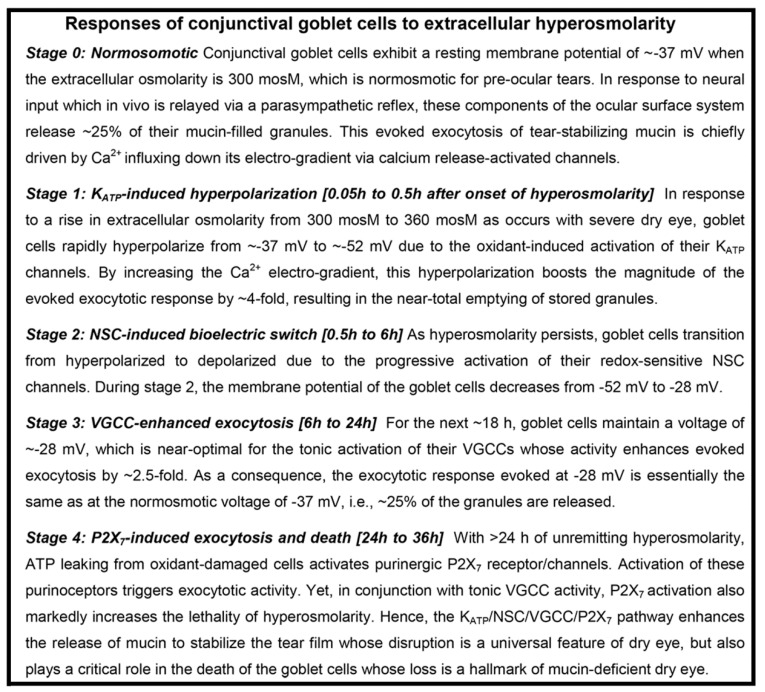
Overview of the response of conjunctival goblet cells to an increase extracellular hyperosmolarity from 300 to 360 mosM, as occurs with highly symptomatic dry eye. This outline incorporates knowledge gained by numerous investigators [2], but focuses on the findings of recent studies using freshly isolated rat conjunctival specimens as an advantageous experimental model for the detection and characterization of hyperosmotic-induced changes in ion channel activity, exocytotic function, and cellular viability [9,10]. With unremitting exposure to a 360-mosM microenvironment, conjunctival goblet cells activate the K_ATP_/NSC/VGCC/P2X_7_ pathway, which boosts exocytotic activity, but ultimately increases the hyperosmotic-induced death of these mucin-releasing cells, whose loss is a histopathologic feature of chronic dry eye [13,14,15]. Abbreviations: K_ATP_, ATP-sensitive K^+^ channel; NSC, nonspecific cation channels; VGCC, voltage-gated calcium channels.

**Figure 2:**
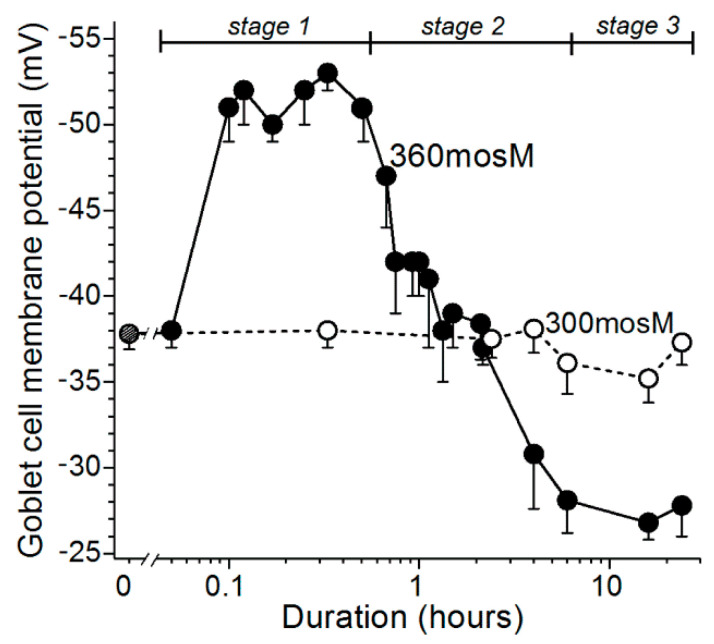
Membrane potentials of goblet cells located in excised conjunctival specimens maintained in a normosmotic 300-mosM solution or a hyperosmotic 360-solution. Data are from recent electrophysiological studies [9,10]; vertical bars show standard errors. During stage 1, goblet cells rapidly hyperpolarize from ~−37 to ~−52 mV (*p* ≤ 0.0001). After ~30 min, the goblet cells begin a ~6-h depolarization that terminates as their membrane reaches −28 mV, which is significantly lower (*p* < 0.0001) than the voltages in stage 1 or under normosmotic conditions (stage 0). Throughout the 18 h of stage 3, goblet cells maintain their membrane potential at ~−28 mV.

Based on experiments using freshly excised rat conjunctiva as an advantageous model for the detection and characterization of hyperosmotic-induced changes in ion channel activity, exocytotic function, and cellular viability [9,10], we now have identified four stages (Figure 1) in the response of conjunctival goblet cells to a rise in extracellular osmolarity from 300 mosM to 360 mosM, as occurs in highly symptomatic dry eye [1]. The first stage is highlighted by the rapid-onset of a ~15-mV increase in the voltage from −37 mV to −52 mV (Figure 2). The oxidant-dependent activation of hyperpolarizing K_ATP_ channels induces this voltage increase, which is functionally important due to the resulting increase in the electro-gradient for Ca^2+^, whose influx via calcium release-activated channels (CRACs) is the chief driver of the exocytotic response to acetylcholine and other neural inputs [8,11,12]. Supporting this scenario, electrophysiological recordings from single goblet cells located in conjunctival specimens demonstrated that the cholinergic-evoked increase in membrane capacitance, which is a quantitative measure of exocytosis, is ~4-fold greater at −52 mV as compared with −37 mV. Calculations further revealed that the massive exocytosis evoked at −52 mV results in the near-total emptying of stored mucin-filled granules [9]. Building on these recent observations, an aim of the present study was to begin to characterize the kinetics for the replenishment of granules after their near-total exocytotic emptying during stage 1.

Our electrophysiological analyses further established that after ~30 min of extracellular hyperosmolarity, goblet cells fundamentally change their bioelectric profile [9,10]. Namely, during stage 2, which extends from ~0.5 h to ~6-h after the onset of hyperosmolarity, conjunctival goblet cells gradually switch from being hyperpolarized to becoming depolarized due to the progressive activation of their redox-sensitive depolarizing NSC channels (Figure 1; [9]). As a result, during stage 2, the membrane potential of the goblet cells steadily declines from −52 mV to −28 mV (Figure 2).

After the NSC-induced depolarization during stage 2 lowers the membrane potential of the goblet cells to −28 mV, these cells enter the third stage of their response to sustained extracellular hyperosmolarity (Figure 1). Throughout the ~18 h of stage 3, the goblet cell voltage is maintained at ~−28 mV (Figure 2). Of functional importance, this voltage is near-optimal for the tonic activation of the goblet cell VGCCs whose activity augments by more than two-fold the calcium-dependent exocytotic response to neural input [10]. As a consequence of the VGCC-induced enhancement of evoked exocytosis, cholinergic input evokes release of ~25% of the stored granules, which, despite depolarization to −28 mV, is essentially the same as observed at the normosmotic membrane potential of −37 mV (Figure 1). In the present study, we assessed the possibility that the activation of VGCCs not only boosts the evoked release of granules, but may also enhance the rate at which granules are replenished after the exocytotic depletion of these mucin-filled packets.

The final stage in the goblet cell response commences after ~24 h of unremitting hyperosmolarity (Figure 1). The key mechanistic feature of stage 4 is the endogenous activation of purinergic P2X_7_ receptors/channels by ATP leaking from osmotically damaged cells [10]. In experiments monitoring the membrane capacitance of single goblet cell located in conjunctiva specimens, we determined that P2X_7_ activation rapidly triggers exocytotic activity [10]. However, over time, our viability assays revealed that activation of these purinoceptors, in conjunction with on-going VGCC activity, markedly increases the vulnerability of conjunctival goblet cells to the lethality of hyperosmolarity [10]. Hence, not only is the release of tear-stabilizing mucin enhanced, but there is a VGCC- and P2X_7_-dependent acceleration in the hyperosmotic-induced death of the conjunctival goblet cells whose loss is a hallmark of dry eye [13,14,15].

To better characterize the functional capability of the goblet cells as they respond to sustained hyperosmolarity, the present study sought to characterize the impact of ion channels on the time course for replenishment of releasable granules after an evoked exocytotic response. Our new findings indicate that the adaptive response of the goblet cells is enhanced when the depolarization-induced activation of their VGCCs accelerates the rate of granule replenishment and thereby boosts their capacity to supply the tear-stabilizing mucin that aids in restoring the microenvironment homeostasis essential for optimal vision.

## 2. Results

An initial objective of this study was to establish the relationship between the voltage of a goblet cell and the magnitude of its evoked exocytotic response. An impact of voltage is relevant to the response of conjunctival goblet cells to hyperosmolarity since their membrane potential increases from −37 mV under normosmotic conditions to −52 mV during stage 1 of their response to a rise in extracellular osmolarity (Figure 2). Subsequently, during stage 2 (0.5 to 6 h of hyperosmolarity), the goblet cell voltage decreases to −28 mV (Figure 2). Furthermore, an effect of voltage on evoked exocytosis is expected since the influx of Ca^2+^ down its electro-gradient via calcium release-activated channels (CRACs) is the chief driver of the exocytotic response of the conjunctival goblet cells to acetylcholine as well as other neural inputs [8,11,12].

**Figure 3:**
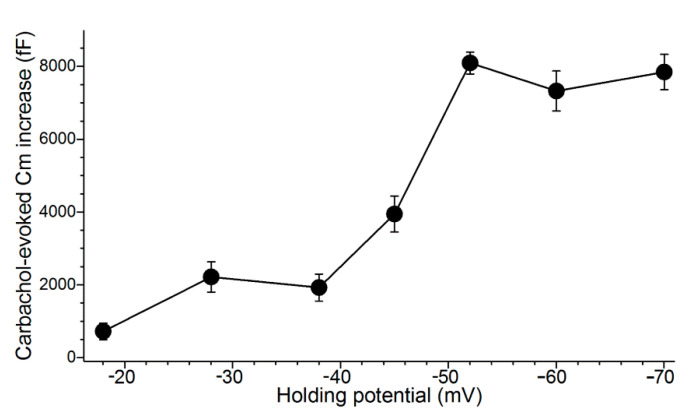
Relationship between goblet cell voltage and the cholinergic-evoked increase in membrane capacitance (Cm). Conjunctival specimens were bathed in the 300-mosM solution which was supplemented for 15 s in with the cholinergic agonist, carbachol (10 µM). Data for holding potentials of −18 and −28 mV are from Puro [10]; for holding potential of −38 mV and −52 mV, the data are from Puro [9]. At the other tested holding potentials, four goblet cells were sampled. Error bars show standard errors. As discussed in the text, the similarity of the carbachol-induced Cm increases observed at −28 mV with that observed at −38 mV is due to the exocytotic enhancing impact at −28 mV of the VGCCs, which are optimally activated at that voltage [10]. Additionally, as discussed in the text, the lack of an increase in evoked Cm at holding potentials more negative than −52 mV supports earlier calculations [9] showing that at −52 mV essentially all of the stored granules of a goblet cell are exocytosed during carbachol exposure. Thus, despite the increased Ca^2+^ electro-gradient at −60 mV and −70 mV as compared with −52 mV, the maximum evoked response is achieved at −52 mV.

To determine the relationship between voltage and the magnitude of evoked exocytosis, we assayed cholinergic-evoked exocytosis in goblet cells located in freshly excised conjunctival specimens (Figure 3).

In these experiments, a perforated-patch pipette sealed onto a goblet cell was used to measure the membrane capacitance (Cm), which is a quantitative indicator of exocytosis. In addition, current passed via the recording pipette maintained the sampled goblet cell at a predetermined holding potential. During these recordings, exocytosis was evoked by supplementing the perfusate with the cholinergic agonist, carbachol (10 µM). As summarized in Figure 3, the carbachol-evoked Cm increase was maximal at voltages of ~−50 mV or more negative. Consistent with previous calculations indicting that cholinergic-evoked exocytosis at −52 mV results in the near-total release of stored granules [9], the carbachol-evoked Cm increases measured in goblet cells maintained at holding potentials of −60 and −70 mV were not significantly larger than the increase observed at −52 mV. Thus, even though the Ca^2+^ electro-gradient was increased, the releasable pool was maxed out at −52 mV. We concluded that the K_ATP_-induced hyperpolarization to −52 mV during stage 1 optimally positions the goblet cell voltage for the maximum release of tear-stabilizing mucin. Hence, a previously unappreciated feature of conjunctival goblet cells is their expression of a sufficient number of K_ATP_ channels to drive their membrane potential to −52 mV and thereby to maximize the exocytotic release of tear-stabilizing mucin.

Figure 3 also reveals that the evoked exocytotic responses at −38 and −28 mV are essentially equal, despite the 10-mV difference in the electro-gradient for Ca^2+^. Indicative that the activation of VGCCs at −28 mV boosts the magnitude of evoked exocytosis, we observed that the Cm increase evoked by the cholinergic agonist carbachol at −28 mV in the presence of the VGCC blocker, nifedipine (2 µM), was only 870 ± 240 fF (*n* = 4), which is significantly less (*p* = 0.0237) than the increase of 1810 ± 200 fF (*n* = 4) observed at −38 mV in the presence of nifedipine [10]. Thus, when VGCCs are blocked, the effect on evoked exocytosis of the difference in the Ca^2+^ electro-gradient at −38 versus −28 mV is revealed. Of relevance to conjunctival goblet cells during stage 3 of their response to hyperosmolarity, this and our previous studies [10] indicate that the VGCCs activated at −28 mV boost the magnitude of evoked exocytosis by more than two-fold.

In additional studies, we sought to ascertain how long it takes for a goblet cell to replenish its exocytotically depleted store of granules. The time-course for granule replenishment is of functional importance since the repetitive release mucin would enhance the adaptive response to dryness/hyperosmolarity. In a series of Cm recordings from single goblet cells located in conjunctival specimens, the releasable pool of granules was assayed at various intervals of times after triggering the exocytotic-induced emptying of granules during the hyperpolarized stage 1. More specifically, the experimental protocol began with an initial 15-s exposure of excised conjunctival specimens to the carbachol-containing 360-mosM solution during the midst of stage 1, i.e., 15 to 25 min after the increase in extracellular osmolarity from 300 to 360 mosM. Subsequently, after the specimen had been bathed in the carbachol-free 360-mosM solution for an additional 0.4 to 3 h, a second 15-s exposure to the carbachol-containing 360-mosM solution was administered while the Cm of a sampled goblet cell was monitored at a holding potential of −52 mV. This Cm assay was performed at a holding potential of −52 mV since, as shown in Figure 3, carbachol-evoked exocytosis at this voltage results in the near-total emptying of stored granules.

**Figure 4:**
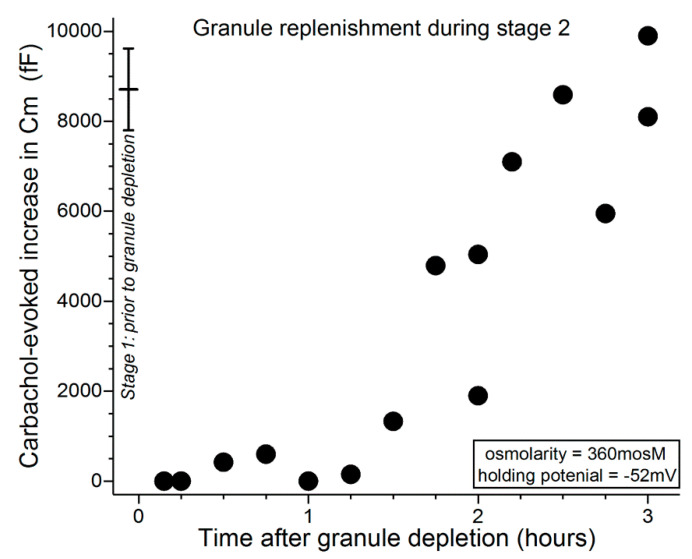
Time course for granule replenishment after near-total exocytotic-induced depletion of granules during stage 1. 15 to 25 min after an increase in extracellular osmolarity from 300 to 360 mosM, conjunctival specimens were exposed for 15 s to the carbachol-supplemented 360-mosM solution. Subsequently, specimens were again exposed for 15 s to the carbachol-containing 360-mosM solution as the Cm of a sampled goblet cell was monitored at a holding potential of −52 mV, which allowed quantification of the releasable pool of granules (Figure 3 [9]). The data point (*n* = 17) at time 0 shows the mean Cm increase (±SD) evoked by an initial exposure to carbachol during stage 1 (from Puro [9]). A massive exocytotic response during stage 1 is initially followed by a ~1-h relative refractory period in which evoked exocytosis is minimal. Subsequently, releasable granules are replenished during the next hour or more.

As demonstrated in Figure 4, when the second carbachol exposure occurred within ~1 h of the initial cholinergic stimulation, the evoked Cm increase was minimal. After this relative refractory period, evoked increases in Cm were detected, and near-complete replenishment was observed 2 to 3 h after the initial massive exocytotic response. These observations indicate that, after massive exocytosis during stage 1, the capacity of conjunctival goblet cells to release tear-stabilizing mucin is minimal for an hour and remains compromised for an additional hour or more.

In other Cm experiments, we focused on the replenishment of granules during the third stage of the response of conjunctival goblet cells to sustained extracellular hyperosmolarity. Notable is that throughout stage 3 (6 h to 18 h of hyperosmolarity), the membrane potential of the goblet cells is maintained at ~−28 mV (Figure 2), which is the voltage at which goblet cell VGCCs are tonically activated [10]. Previously, we discovered that this VGCC activity boosts evoked exocytosis [10]. In the present study, we tested the additional possibility that VGCCs also play a role in the replenishment of granules after exocytosis. We considered this scenario since replenishment of releasable vesicles/granules in some well-studied exocytotic systems occurs via a calcium-sensitive mechanism [16,17].

To begin to test the hypothesis that VGCCs play a role in the replenishment of mucin-filled granules during stage 3, we employed an experimental protocol using conjunctival specimens in which goblet cells during stage 3 of their response to hyperosmotic were initially depleted of most of their granules. Subsequently, the replenishment of releasable granules was assayed at −52 mV, which, as noted (Figure 3), is a voltage at which evoked exocytosis is maximal. More specifically, in conjunctival specimens maintained for 6 to 18 h in the 360-mosM solution, i.e., during stage 3 (Figure 1 and Figure 2), the exocytotic depletion of granules was induced by three 15-s exposures at 4-min intervals to the carbachol-containing 360-mosM solution. Confirming the exocytotic impact of this protocol, electrophysiological recordings made at the holding potential of −28 mV (the resting membrane potential of the conjunctival goblet cells in stage 3) showed that this 3-exposure protocol evoked a cumulative Cm increase of 8350 ± 570 fF (*n* = 4), which calculations indicate represents the near-total release of stored granules [9]. To begin to characterize the rate of granule replenishment after administration of this depletion protocol, conjunctival specimens were again exposed for 15 s to carbachol as goblet cell Cm was monitored at a holding potential of −52 mV.

**Figure 5:**
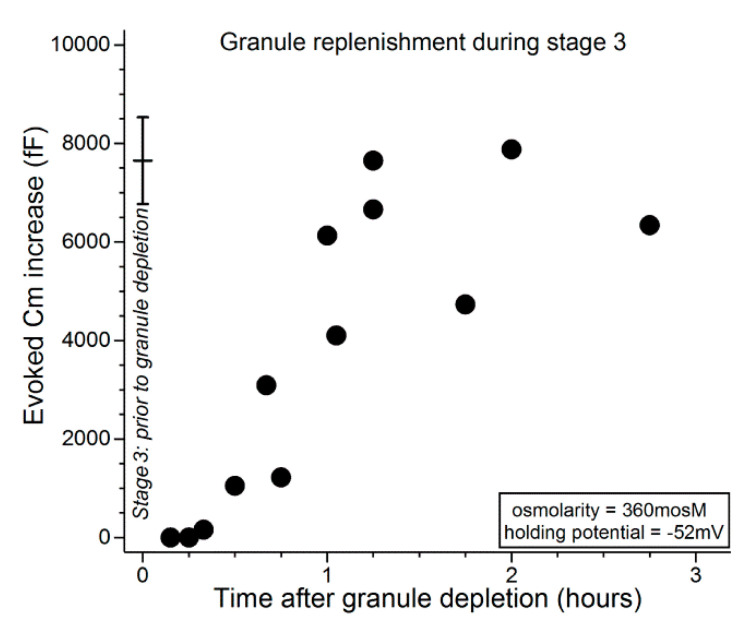
Time course for the replenishment of releasable granules during stage 3. At various times after administration of a granule-depleting protocol (see text), conjunctival specimens were again exposed for 15 s to the carbachol-containing 360-mosM solution as the Cm of a sampled goblet cell was monitored at −52 mV (this holding potential allowed assessment of the size of the pool of releasable granules). The data point at time 0 shows the mean (±SD; *n* = 4) for the evoked Cm increase measured in stage 3 during a first exposure to carbachol. In stage 3, granule replenishment begins ~30 min after the exocytotically induced emptying of stored granules. After an additional ~45 min, goblet cells achieve their full complement of granules.

Figure 5 shows that during the initial ~30 min after administration of the depletion protocol, carbachol exposure evoked minimal, if any, increase in Cm. In experiments in which the interval between depletion and subsequent exposure to carbachol was lengthened, Cm increases were evoked and over time approached a level reflective of near-complete replenishment of releasable granules (Figure 5). Consistent with VGCCs playing a role during stage 3, comparison of the data in Figure 5 with those in Figure 4 indicates that granule replenishment is more rapid during stage 3, when the VGCCs are tonically activated. Namely, the Cm increases evoked 1 to 2 h after granule depletion were 6190 ± 620 fF (*n* = 6; holding potential = −52 mV) and 2200 ± 910 fF (*n* = 6; *p* = 0.0047) in stages 3 (after depletion earlier in stage 3) and 2 (after depletion in stage 1), respectively.

To more convincingly assess the impact of VGCCs on granule replenishment during stage 3, Cm assays were performed in the presence of nifedipine (2 µM), which blocks these channels in the goblet cells [10]. We observed that, 1 to 2 h after administration of the granule depletion protocol, the carbachol-evoked Cm increase in the presence of nifedipine was only 1450 ± 205 fF (*n* = 4; holding potential = −52 mV), which was less (*p* = 0.0003) than the 6190 ± 620 fF (*n* = 6; holding potential = −52 mV) increase evoked in the absence of nifedipine. These findings indicate that a nifedipine-sensitive mechanism markedly accelerates granule replenishment during stage 3.

Taken together, these observations along with our previous findings [10] indicate that VGCC activation during the stage 3 of the goblet cell response to hyperosmolarity not only boosts cholinergic-evoked exocytosis, as we recently reported [10], but the activity of these Ca^2+^-permeable pathways also accelerates granule replenishment. Due to the VGCC-induced enhancement of the exocytotic release of granules and the subsequent replenishment of granules, we propose that the adaptive effectiveness of the conjunctival goblet cells to persistent hyperosmolarity is enhanced. Yet, our investigations have also revealed that conjunctival goblet cells undertake significant risk when their VGCCs become activated since the activity of these channels increases the vulnerability of the conjunctival goblet cells to the toxicity of hyperosmotic-induced oxidants [10].

## 3. Discussion

The goblet cells of the conjunctiva play a key role in establishing and maintaining the tear film, which constitutes a functional interface between the eye and the external environment and thereby is essential for the optimization of visual function. By releasing mucin whose water-binding capacity creates a muco-aqueous gel that stabilizes the tear film, these cells contribute importantly to the adaptive response of the ocular surface system to dry eye, which is a common, vision-impairing, painful disorder characterized by evaporation-induced hyperosmolarity of the pre-ocular fluid. Neural reflexes triggered by dryness/hyperosmolarity cause the goblet cells of the conjunctiva to exocytotically release mucin, which aids in restoring the normal extracellular microenvironment. Despite important advances in understanding the mechanisms by which conjunctival goblet cells transduce neural input into the exocytotic output of mucin [2], knowledge of how ion channels impact this adaptive response remains limited.

In the first patch-clamp studies of goblet cells located within the conjunctiva, we discovered that these mucin-releasing components of the ocular surface system express a variety of functional ion channels [9,10]. Further, we found that during sustained extracellular hyperosmolarity, which is a universal feature of dry eye, goblet cells of the conjunctiva sequentially activate a series of ion channels that robustly modulate the exocytotic response to neural inputs, such as acetylcholine. The ion channels activated during the course of sustained hyperosmolarity are: hyperpolarizing K_ATP_ channels, depolarizing nonspecific cation (NSC) channels and depolarization-activated voltage-gated calcium channels (VGCCs), as well as purinergic P2X_7_ receptor/channels [9,10].

Although increased osmolarity of the tear film is a well-recognized universal feature of clinical dry eye [1], an unresolved issue has been whether hyperosmolarity is a critical pathophysiological event in the mucin depletion that leads to the chronic form of this disorder. Indicative that extracellular hyperosmolarity does play a key role, this and our previous experimental studies [9,10] reveal that an osmotic increase initiates ion channel-dependent mechanisms that potently boost the depletion of mucin stores and, ultimately, the death of the mucin-releasing goblet cells.

Based on our analysis of ion channel activity and exocytotic function in goblet cells located in freshly excised rat conjunctiva [9,10], we now identify four stages in the adaptive response of these cells to the sustained elevation in the extracellular osmolarity to 360 mosM (Figure 1), which is associated with highly symptomatic dry eye [1]. During the first stage, oxidant-activated K_ATP_ channels rapidly generate a ~15-mV voltage that increases the membrane potential from ~−37 to ~−52 mV. In turn, this hyperpolarization markedly boosts the magnitude of the exocytotic response to cholinergic input and result in the near-total emptying of mucin-filled granules. In our experimental preparation of freshly excised rat conjunctiva, the ~30-min of K_ATP_-induced hyperpolarization is followed by stage 2, in which the goblet cells fundamentally switch their bioelectric profile from hyperpolarized to depolarized. This bioelectric switch is mediated over a ~6 h period of continued hyperosmolarity as the progressive activation of oxidant-sensitive depolarizing NSC channels steadily lowers the membrane potential. After ~6 h in stage 2, the goblet cell voltage is −28 mV, which is subsequently maintained throughout the 18 h of stage 3. A key functional impact of the NSC-induced depolarization is that, at −28 mV, the voltage is optimal for the tonic activation of the goblet cell VGCCs whose opening enhances evoked exocytosis [10]. Stage 4 commences when hyperosmolarity persists for >6 h. A highlight of this final stage is activation of purinergic P2X_7_ receptor/channels by ATP released from osmotically damaged cells. With the activation of these purinoceptors, exocytotic activity is triggered, but over time, P2X_7_ activation, in conjunction with VGCC activity, renders the goblet cells highly vulnerable to the lethality of hyperosmotic-induced oxidants. Hence, our experimental findings indicate that the goblet cells of the conjunctiva respond to unremitting hyperosmolarity by eventually adopting a high-risk adaptive strategy. The K_ATP_/NSC/VGCC/P2X_7_ pathway not only boosts the release of tear-stabilizing mucin, but if a return to normosmolarity does not occur, then this pathway profoundly compromises the viability of the conjunctival goblet cells whose loss is a hallmark of chronic mucin-deficient dry eye.

To more fully characterize the exocytotic activity of the conjunctival goblet cells, we used patch-clamp recordings to assess the rate at which these cells replenish their supply of releasable granules after the near-total exocytotic-induced emptying of these mucin-filled packets. High resolution monitoring of the membrane capacitance (Cm), which is a measure of exocytosis, revealed that the massive exocytosis evoked by cholinergic input during the hyperpolarized stage 1 is followed by a ~1-h relative refractory period in which minimal, if any, exocytosis can be evoked by neural input (Figure 4). Cm measurements further demonstrated that the replenishment of releasable granules takes additional hours. Notable is that this relatively protracted time course is consistent with morphological observations of the recovery of non-conjunctival goblet cells in vivo after granule emptying [18,19]; there appears to be no comparable anatomical study of conjunctival goblet cells.

**Figure 6:**
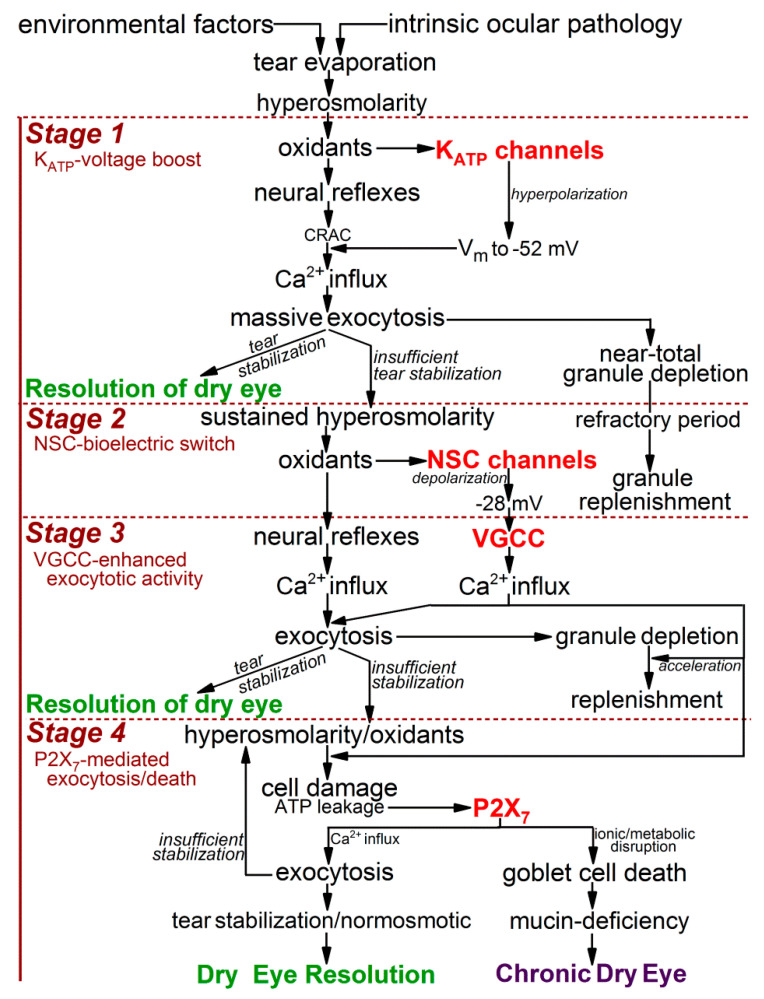
Model of the impact of the K_ATP_/NSC/VGCC/P2X_7_ pathway on the adaptive physiologic and lethal pathobiologic responses of conjunctival goblet cells to the extracellular hyperosmolarity associated with severe dry eye. Details in text. Abbreviations: CRAC, calcium release-activated channels; K_ATP_, ATP-sensitive K^+^ channel; NSC, nonspecific cation channels; P2X_7_, purinergic P2X_7_ receptor/channels; VGCC, voltage-gated calcium channels; Vm, membrane potential.

Our working model incorporating the emerging concepts of how ion channels impact the physiological and pathobiological responses of conjunctival goblet cells to dryness/hyperosmolarity is illustrated in Figure 6.

We propose that the exocytotic release of a massive bolus of mucin during stage 1 provides an adaptive response that is likely to be particularly effective in ameliorating the pre-ocular dryness/hyperosmolarity commonly caused by transient ‘everyday’ environmental conditions such as blowing air (wind). Yet, if the cause of tear instability/evaporation is chronic pathologic disorders such as lacrimal/ocular gland dysfunction, then the ‘shock and awe’ of a massive mucin bolus is likely to be insufficient for maintaining long-term normosmolarity. In fact, the emptying of mucin-filled granules in stage 1 leaves the goblet cells functionally compromised for hours.

Our electrophysiological findings indicate that if extracellular normosmolarity is not restored by the ocular surface system during stage 1, then the conjunctival goblet cells undergo a fundamental change in their bioelectric profile. During the subsequent 6 h of stage 2, goblet cells switch from being hyperpolarized at a membrane potential of ~−52 mV to becoming depolarized with a voltage of ~−28 mV, which is ~10 mV less negative than the normosmotic membrane potential of ~−37 mV (Figure 2). By switching to depolarization, goblet cells acquire functional benefits provided by the activation of their VGCCs whose tonic activity is near-maximal at −28 mV [10], which is the goblet cell voltage throughout the ~18 h of stage 3 (Figure 2). Of functional importance, VGCC activity during stage 3 not only increases the exocytotic response to neural input by ~2.5-fold [10], it also (Figure 4 and Figure 5) accelerates granule replenishment by ~2-fold. By these VGCC-dependent mechanisms, conjunctival goblet cells become better adapted to repetitively release tear-stabilizing mucin and thereby to contribute to the resolution of dryness/hyperosmolarity induced by persistent evaporation-enhancing environmental and intrinsic conditions. Yet, if normosmolarity is not restored during stage 3, then the goblet cells progress to the high-risk stage 4 in which the endogenous activation of P2X_7_ receptor/channels provides mucin to the ocular surface, but in conjunction with the activity of VGCCs, also markedly boosts the vulnerability of the goblet cells to the oxidant-mediated lethality of hyperosmolarity [10].

Our experimental model of freshly excised rat conjunctiva has allowed us to perform the first patch-clamp analyses of goblet cell physiology and pathobiology [9,10]. However, a caveat of this model is that conclusions derived from ex vivo specimens ultimately require in vivo confirmation. However, at present, this confirmation does not appear feasible since the monitoring of goblet cell voltage and membrane capacitance in vivo awaits methodological advances. On the other hand, use of excised specimens has permitted the first electrophysiological studies of single goblet cells located within their intrinsic tissue, rather than after enzymatic tissue dissociation. Furthermore, our experimental preparation has facilitated the first use of the powerful “sine + DC” technique to obtain high-resolution Cm measurements of the evoked exocytotic activity of single goblet cells in any tissue. This biophysical approach has revealed the impact of ion channels on goblet cell exocytotic activity, including the release and replenishment of mucin-filled granules. In addition, this Cm assay has also demonstrated that the amount of stored granules released in response to neural input is highly dependent on the transmembrane voltage, which is markedly altered as hyperpolarizing K_ATP_ and depolarizing NSC channels become activated during sustained hyperosmolarity. In addition, use of this experimental preparation allowed us to establish that the size of the evoked exocytotic response is also modulated by the Ca^2+^-permeable VGCCs and P2X_7_ receptor/channels.

Despite the opportunities provided by the experimental model of excised conjunctival specimens, it is important to note that the lack blood flow and intrinsic neural input to these specimens deprives goblet cells and their conjunctival neighbors of putative nutritional, trophic, and other molecules that may modulate ion channel function, exocytotic activity, and cellular viability. Thus, future experimental work is needed to ascertain whether the hyperosmotic-induced death of goblet cells occurs more rapidly in the excised conjunctival specimens than in the in vivo conjunctiva where the ocular vasculature, innervating nerves, immuno-molecules, and anatomical structures such as the lids may exert protective roles that delay or the death of conjunctival goblet cells during unremitting hyperosmolarity. Another caveat for use of our experimental model is that, although our ultimate aim is to elucidate the pathophysiology of dry eye in humans, the rare availability of appropriate human conjunctival specimens led us to study goblet cells of the rat conjunctiva. Yet, it is reassuring that despite goblet cells in the rat conjunctiva being located in clusters, whereas these mucin-releasing cells in the human conjunctiva are solitary, conjunctival goblet cells of rats and humans share important morphological and functional attributes [20,21,22]. In the future, studies will need to establish that the K_ATP_/NSC/VGCC/P2X_7_ pathway also plays a role in the physiology and pathobiology of the goblet cell in the human conjunctiva. On the other hand, it should be noted that the ability to apply high-temporal resolution monitoring of the membrane capacitance to the analysis of the exocytotic activity of single goblet cells located within their intrinsic tissue now opens the possibility of more completely elucidating the exocytotic mechanisms used by these components of the ocular surface system to help maintain the tear film homeostasis required for optimal visual function.

In summary, building upon recent electrophysiological analyses of single goblet cells located in the conjunctiva or in any other tissue, this study now reveals that the multi-hour process by which mucin-filled granules are replenished after their exocytotic depletion is accelerated by ~2-fold when the NSC-induced depolarization to −28 mV causes the goblet cell VGCCs to become tonically activated. In turn, due to the VGCC-induced acceleration of granule replenishment, as well as the VGCC-mediated increase in evoked exocytosis, the switch in the bioelectric profile of the goblet cells from hyperpolarized in stage 1 to depolarized in stage 3 is a previously unappreciated adaptive response that serves to enhance the supply of extracellular mucin when the microenvironment is hyperosmotic. Yet, this adaptive strategy carries risk since tonic VGCC activity plays a synergistic role with the P2X_7_ receptor/channels activated in stage 4 to accelerate the hyperosmotic-induced death of the conjunctival goblet cells whose loss is a histopathological feature of irreversible mucin-deficient dry eye.

## 4. Materials and Methods

Experimental protocols for animal use were approved by the Institutional Animal Care and Use Committee of the University of Michigan (Protocol: PRO00008287 approved 18 April 2018) and were consistent with the Code of Practice for the Housing and Care of Animals Used in Scientific Procedures. Sprague-Dawley and Long-Evans (Charles River, Cambridge, MA, USA) rats were kept on a 12-h alternating light/dark cycle and provided food and water ad libitum. Approximately equal numbers of males and females were used.

### 4.1. Experimental Preparation

Conjunctival specimens were prepared as detailed elsewhere [9,10]. In brief, immediately after a rising concentration of carbon dioxide resulted in death of 8- to 26-wk-old rats, ~7 by ~5-mm conjunctival specimens were excised from each eye, hemi-sectioned, positioned with the exterior surface upwards in a glass-bottom 0.75-mL recording chamber and stabilized with a harp-shaped tissue anchor (SHD26GH/10; Warner Instruments, Hamden, CT, USA). The chamber was then filled with a solution consisting of 105 mM NaCl, 15 mM NaHCO_3_, 10 Na-Hepes, 20 mM KCl, 0.5 mM CaCl_2_, 0.5 mM MgCl_2_, and 3 mM glucose; the pH was adjusted to 7.5, and a vapor pressure osmometer (Vapro 5600, Wescor, Logan, UT, USA) confirmed that osmolarity was 300 mosM, which is normosmotic for the tears of rats, rabbits, and humans [23,24,25]. Additionally of note, the K^+^ and divalent cation concentrations, as well as the pH, reflect the values measured in normal tears of various animals and humans [1,23,24,25,26]. Prior to experimentation, specimen-containing chambers were kept in a Billups–Rothenberg modular chamber (Billups-Rothenberg, Inc., Del Mar, CA, USA) at 100% humidity and 22–23 °C. Confirming that maintenance in these modular chambers was not associated with a significant osmotic change, we determined that after 24 h, the osmolarity of the bathing solution remained within 4% (*n* = 7) of the initial value.

#### 4.1.1. Electrophysiology

**Figure 7:**
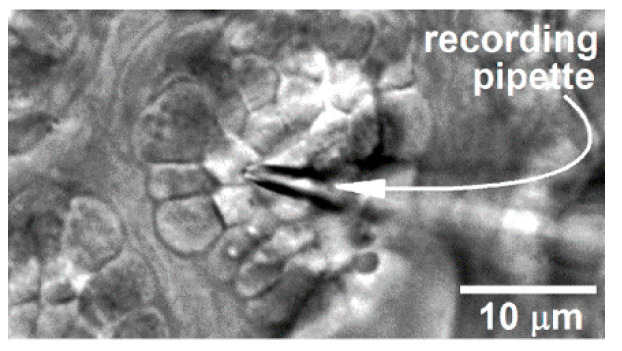
Differential interference contrast/infrared image of a portion of a freshly excised rat conjunctiva in which a perforated-patch pipette is sealed onto a goblet cell located within a complex of these mucin-releasing cells. With permission from Puro [9].

As described previously [9,10], a specimen-containing chamber was positioned onto the stage of an upright microscope and viewed at 400X with differential interference contrast/infra-red optics. The recording chamber could be perfused at ~2 mL/min with either the 300-mosM solution described above or a 360-mosM solution that included the non-NaCl components of the 300-mosM solution plus an appropriate concentration of NaCl to achieve 360 mosM, which was confirmed by vapor pressure osmometry (Vapro 5600). Care was taken to obtain electrophysiological recordings from multi-cellular clusters in which all of the goblet cell were viable. Hence, as detailed previously [10], each conjunctival specimen was exposed for precisely 15 min to the 300-mosM supplemented with 0.04% trypan blue, whose presence within a cell is indicative of death. After the specimen was washed 3 times, it was examined with bright-field optics at X100 magnification in order to assess goblet cell positivity for trypan blue. Locations of goblet cell complexes containing exclusively trypan blue-negative cells were documented using the locale’s x–y coordinates on the microscope stage, as well as a sketch and/or a digital image of the location within the specimen. Using 5 to 10 MΩ pipettes (TW150F-4, World Precision Instruments, Sarasota, FL, USA) filled with a solution consisting of 50 mM KCl, 65 mM K_2_SO_4_, 6 mM MgCl_2_, 10 mM K^+^-Hepes, 60 μg·mL^−1^ amphotericin B, and 60 μg·mL^−1^ nystatin at pH 7.35 and 280 mosM, perforated-patch recordings were obtained from the apical surface of cells located in a goblet cell cluster, which is a well-characterized and easily identified feature of the rat conjunctiva ([22,27]; Figure 7).

**Figure 8:**
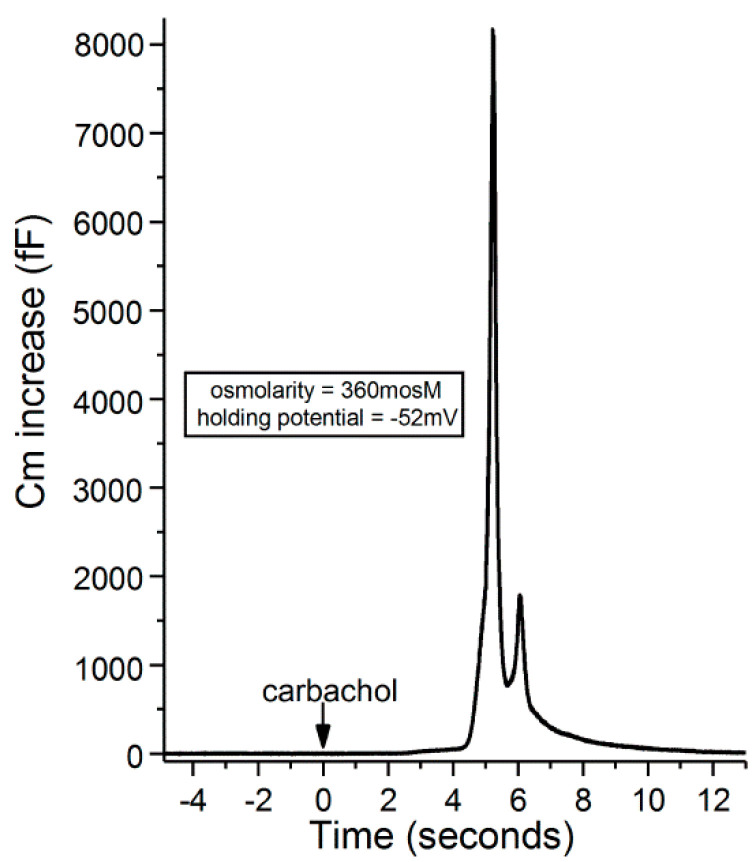
Recording of a carbachol-induced increase in the membrane capacitance of a goblet cell located in an excised conjunctival specimen exposed for 20 min to the 360-mosM. The holding potential of the sampled goblet cell was −52 mV. With permission from Puro [9].

Recordings with access resistances of <25 MΩ and <10% variability were made via an EPC-9 (HEKA Elekronik, Lambrecht, Germany) patch-clamp amplifier, which used Patchmaster software (HEKA) to control data sampling and acquisition. Data analysis was also aided by graphics software (Origin 2018, OriginLab Corp, Northampton, MA, USA). Adjustment was made off-line for the junction potential, which was −8 mV for the solutions used in this study. Evoked exocytosis was quantified by monitoring the membrane capacitance (Cm) of single goblet cells, as described in earlier studies [9,10] and illustrated in Figure 8. Although a Cm increase reflects net exocytotic and endocytotic activities, the increase in Cm detected soon after exposure to an agonist chiefly reflects the initially evoked exocytosis preceding the onset of endocytotic activity [28]. Cm measurements were obtained by using the “sine + DC” method [29] in which a 30-mV peak sinusoidal signal was applied at a frequency 1.8 kHz to a voltage-clamp recording obtained via a perforated-patch pipette, which also was used to inject current that maintained the voltage of the sampled goblet cell at a pre-selected holding potential. With Patchmaster software (HEKA) emulating a lock-in amplifier, the resulting current response was used to calculate Cm [30]. Recordings with spontaneous fluctuations of ≥100 fF were excluded.

In Cm recordings to assess the size of the releasable pool of stored granules during granule replenishment, sampled goblet cells were monitored at a holding potential of −52 mV since the near-total emptying of stored granules occurs at this voltage (Figure 3; [9]). For each recording, the cholinergic-evoked Cm was the maximum increase in Cm observed ≤10 s after the onset of a 15-s exposure to the 360-mosM solution supplemented with 10 µM carbachol. Indicative that 10 µM is a maximally effective concentration of carbachol, Cm increases evoked in goblet cells sampled at −52 mV during stage 1 during exposure to 10 µM, 25 µM and 50 µM carbachol were 8710 ± 550 fF (*n* = 17; data from Puro [9]), 7860 ± 550 fF (*n* = 3) and 8280 ± 620 (*n* = 3), respectively; these Cm increases were not significantly different.

#### 4.1.2. Chemicals

Chemicals were from Sigma (St. Louis, MO, USA).

#### 4.1.3. Statistics

Data in Figure 2 and Figure 3 are given as means ± SE; ‘*n*’ indicates the number of sampled cells. Probability was evaluated by Student’s two-tailed *t*-test with equal or unequal variance as appropriate. For comparison of two groups, *p* ≥ 0.05 indicated failure to detect a significant difference; the Bonferroni correction was used to adjust the *p*-value for significance when more than two groups were compared.
